# Preliminary Evaluation of 3D-Printed Alginate/Gelatin Scaffolds for Protein Fast Release as Suitable Devices for Personalized Medicine

**DOI:** 10.3390/biomedicines13061365

**Published:** 2025-06-02

**Authors:** Benedetta Ghezzi, Ruben Foresti, Luisa Pia Scialoia, Maddalena Botti, Arianna Mersanne, Fulvio Ratto, Francesca Rossi, Chiara Martini, Paolo Perini, Elda Favari, Antonio Freyrie

**Affiliations:** 1 Department of Medicine and Surgery, University of Parma, Via Gramsci 14/A, 43126 Parma, Italy; 2 Center of Dental Medicine, University of Parma, Via Gramsci 14/A, 43126 Parma, Italy; 3 Institute of Materials for Electronics and Magnetism, National Research Council, Parco Area delle Scienze 37/A, 43124 Parma, Italy; 4 Center of Excellence for Toxicological Research (CERT), University of Parma, Via Gramsci 14/A, 43126 Parma, Italy; 5 Department of Veterinary Science, University of Parma, Via del Taglio 10, 43126 Parma, Italy; 6 Vascular Surgery, Department of Medicine and Surgery, University of Parma, Via Gramsci 14/A, 43126 Parma, Italy; 7 Vascular Surgery, Cardio-Thoracic and Vascular Department, University-Hospital of Parma, Via Gramsci 14, 43126 Parma, Italy; 8 Istituto di Fisica Applicata “Nello Carrara”, Consiglio Nazionale delle Ricerche, 50019 Sesto Fiorentino, Italy; 9 Diagnostic Department, University-Hospital of Parma, Via Gramsci 14/A, 43126 Parma, Italy; 10 Strategic Steering Commitee, Centro Studi SAPIS Foundation, Italian National Federation of Orders of Radiographers and Technical, Rehabilitation, and Prevention Health Professions Research Centre, Via Magna Grecia, 30/A, 00183 Rome, Italy; 11 Department of Food and Drug, University of Parma, Parco Area delle Scienze 27/A, 43124 Parma, Italy

**Keywords:** bioprinting, scaffold, vascular surgery, personalized medicine, atherosclerosis

## Abstract

**Background/Objectives:** Drug-coated balloons (DCBs) are emerging as a promising treatment for peripheral artery disease; however, current technologies lack flexibility in customizing drug release profiles and composition, limiting their therapeutic potential. This study aims to develop a Gelatin (Gel) and Sodium Alginate (Alg) bioink loaded with apolipoprotein A-I (apoA-I) for controlled drug delivery by using additive manufacturing technologies. **Methods**: We developed and printed via rapid freeze prototyping (RFP) a Gel and Alg bioink loaded with different concentrations of apoA-I. Mechanical properties related to compressional and tensile forces have been studied, as well as the structural stability and active release from the 3D structure of apoA-I (cholesterol efflux assays). The biological behavior of HUVEC cells with and without ApoA-I was assessed by proliferation assay, metabolic activity analysis, and fluorescence imaging. **Results**: The 3D structures presented breakpoint stress values consistent with the mechanical requirements for integration within a DCB, and the ability to effectively promote cholesterol transport in J774 cells. Moreover, in vitro studies on HUVECs revealed that the scaffolds exhibited no cytotoxic effects, leading to increased ATP levels and enhanced metabolic activity over time, confirming the possibility to obtain RFP-printed Alg/Gel scaffolds able to provide a stable structure capable of controlled apoA-I release. **Conclusions**: These findings support the potential of Alg/Gel+apoA-I scaffolds as biocompatible drug delivery systems for vascular applications. Their ability to maintain structural integrity while enabling controlled biomolecular release positions them as promising candidates for personalized cardiovascular therapy, facilitating the rapid customization of bioprinted therapeutic platforms.

## 1. Introduction

Cardiovascular diseases (CVDs) currently represent the leading cause of death worldwide. In particular, atherosclerosis is a major underlying cause in many CVDs, accounting for approximately 50–60% of cases, including coronary artery disease, cerebrovascular disease, and aortic and peripheral artery diseases [1,2]. These diseases are among the most widespread, causing an estimated 19.1 million deaths in 2020, with this number projected to rise to 23 million by 2030 [3,4,5].

Atherosclerotic plaques may continually grow and become complicated, causing haemodynamic stenoses or even occlusions, which can result in ischemic organ damage depending on the location of these lesions [5]. Two main treatments for atherosclerotic CVD revascularization are currently available: bypass surgery (BS) and endovascular treatment (EVT), mainly by means of balloon angioplasty (BA). Since BS is associated with higher rate of post-operative morbidity and greater length of stay, BA is often the preferred method to treat these patients [6]. In fact, BA is associated with a less complicated post-operative period, even though with possible lower durability [7,8]. 

Significant progress has been made in endovascular technologies, with ongoing research focused on enhancing mid- and long-term patency rates while maintaining the minimally invasive nature of balloon angioplasty. For example, drug-coated balloon (DCB) angioplasty seems to provide a significant advantage in terms of durability and effectiveness [9,10,11]. The concept of a drug-coated balloon (DCB) for lesion-specific drug delivery was introduced in 2001. More recently, the emergence of next-generation DCB with better drug delivery has sparked renewed interest in this technology and its promise of percutaneous coronary intervention without leaving anything permanently behind [9].

Drug-coated balloons, which represent one of the most significant innovations in EVT, are commonly used to manage these conditions, but there is still lack of effective control of drug release times and compositions. These devices combine the mechanical benefits of balloon catheter dilation with localized delivery of antiproliferative drugs, thus reducing the risk of restenosis and improving long-term clinical outcomes. Reconstituted high-density lipoprotein (rHDL), a formulation of apoA-I, is a promising therapeutic agent in CVDs due to its ability to enhance reverse cholesterol transport (RCT), a key antiatherogenic process [12]. rHDL mimics the function of natural HDL, promoting cholesterol efflux from macrophages in atherosclerotic plaques and facilitating its transport to the liver for excretion. Studies have shown that rHDL infusions can improve endothelial function, reduce inflammation, and stabilize atherosclerotic plaques, thereby reducing the risk of cardiovascular events [13]. In particular, clinical trials using rHDL formulations, such as CSL112, demonstrated significant increases in cholesterol efflux capacity and potential to reduce the burden of coronary artery disease (CAD) in high-risk patients [14]. Despite these benefits, further large-scale trials are required to confirm the long-term efficacy and safety of rHDL therapies in reducing cardiovascular morbidity and mortality. The results of the AEGIS-II trial, despite not achieving the primary endpoint, along with the favorable safety profile and indications of reduced cardiovascular death and non-type 2 myocardial infarctions, may support further investigations into targeted therapies involving apoA-I or rHDL. Specifically, these findings could justify exploring in situ apoA-I/rHDL release as a novel strategy for enhancing cholesterol efflux and stabilizing atherosclerotic plaques in patients with high cardiovascular risk [15], but the absence of a platform (biomaterials and technologies) useful for its management (time and in situ release) must be overcome.

Sodium alginate and gelatin are biocompatible and biodegradable materials widely used for biomedical applications to form hydrogels suitable for cell encapsulation and drug delivery [16,17,18]. Sodium alginate is a natural polysaccharide derived from the cell walls of brown algae, consisting of mannuronic acid (M) and guluronic acid (G) monomers [19,20]. One of its key properties is its ability to form hydrogels via ionic crosslinking with divalent cations such as calcium (Ca^2+^), providing a tunable gelation process [21]. However, alginate lacks cell-adhesive motifs, requiring modifications or blending with other biomaterials to enhance cellular interactions. Gelatin, derived from collagen, is a natural biopolymer widely used in biomedical applications due to its inherent bioactivity and ability to promote cell adhesion, proliferation, and differentiation [22]. It exhibits thermosensitive properties, forming hydrogels at low temperatures and liquefying at physiological conditions, making it highly suitable for bioink formulations [17]. Additionally, gelatin can be crosslinked enzymatically, chemically, or through photocrosslinking (e.g., gelatin methacrylate, GelMA), allowing for improved mechanical stability and controlled degradation rates [16].

Alginate-based hydrogels have been extensively utilized in 3D printing due to their shear-thinning behavior, allowing for smooth extrusion and rapid post-printing gelation, and providing an optimal balance between printability, structural integrity, and functionality [18,23]. Moreover, bioprinting encompasses all processes aimed at combining cells, growth factors, and/or biomaterials to create biomedical structures, often with the goal of replicating the characteristics and morphology of natural tissue [24,25], enabling the rapid and precise customization of personalized devices and drugs to support the restoration of tissue functionalities [26]. Therefore, caused by the expensive customization of the DCB devices [27], this approach may play a pivotal role in producing suitable biomaterial scaffolds for specific applications [24,28,29,30], and, by using dedicated material extrusion techniques, might lead to the production of a composite and biocompatible scaffold [31,32,33] able to quickly release [34] high-density lipoprotein filler with specific concentrations.

The primary objective of this study was to evaluate the effective scalability, in terms of biocompatibility, mechanical properties, and release ability, of 3D-printed scaffolds loaded with Apolipoprotein A-I, obtained by using rapid freeze prototyping (RFP) technology. Biocompatibility tests (MTT, CellTiter-GLO and cell counting) were performed on human endothelial cells (HUVEC). Subsequently, the scaffolds were compressed and stretched to identify the maximum load reachable with the selected non-toxic formulation. Then, apoA-I release was studied through cholesterol efflux assay in murine macrophages (J774), and the achieved results confirmed that this approach is a suitable candidate for the development of customized devices, such as implantable and biodegradable bioprinted hydrogel scaffolds for protein release.

## 2. Materials and Methods

### 2.1. Bioink Development

The identified biomaterials for the scaffold’s fabrication, and useful for vascular applications, was developed selecting medical-grade powders: Sodium Alginate (Alg—W201502, Sigma Aldrich, St. Louis, MO, USA) and Gelatin from porcine skin (Gel—G1890, Sigma Aldrich), with and without apolipoprotein A-I from human plasma (apoA-I, A0722, Sigma Aldrich). Briefly, to obtain the bioink, 2% *w*/*v* of sterile sodium alginate and 10% *w*/*v* of sterile gelatine were vigorously mixed into culturing medium (DMEM High Glucose, Dominique Dutscher, Bernolsheim, France). The 2% (*w*/*v*) Sodium Alginate content was selected based on the minimum value commonly reported in the literature for bioink formulations in tissue engineering applications [35,36]. Preliminary tests showed that lower concentrations resulted in poor construct handling, reduced printability, and inadequate shape retention and confirmed that a 2%(*w*/*v*) Sodium Alginate solution provided the best compromise between fluidity and mechanical stability. The addition of 10% (*w*/*v*) Gelatin from porcine skin further improved the rheological properties [36,37].

The solution obtained was mixed until it became homogeneous and subsequently once an hour for 6 h to allow the complete dissolution of the gelatine. Three experimental concentrations of apoA-I (2.5 µg/mL, 5 µg/mL and 10 µg/mL) were added to the bioink, while 2% alginate and 10% gelatine bioink without apoA-I were used as control. After the overnight incubation, the bioinks were collected through a 5 mL syringe with a 25G needle and used for the 3D-printing processes, and after printing gelled with 50 mM Calcium Chloride (CaCl_2_, C3306, Sigma Aldrich, St. Louis, MO, USA) to ensure an optimal balance between biocompatibility and structural integrity of the scaffold, and preventing excessive crosslinking, which could negatively impact porosity, flexibility, and drug release dynamics [38].

### 2.2. 3D Printing

**Figure 1:**
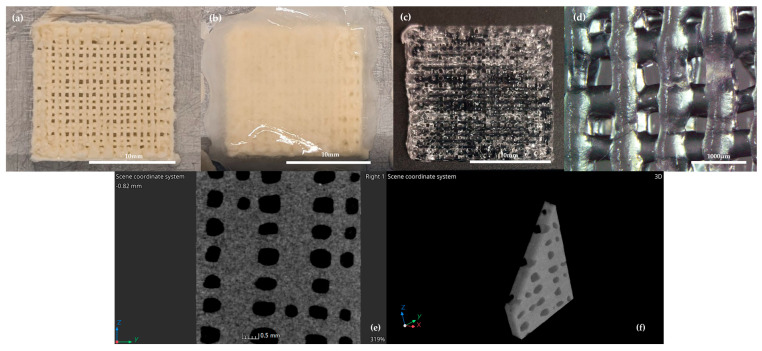
(**a**) Alg/Gel scaffold after the 3D–printing process onto the Peltier cell; (**b**) during the initial freezing and gelification process with CaCl_2_ 50mM and (**c**) after gelification process with CaCl_2_ 50 mM; (**d**) microphotograph of Alg/Gel scaffold structure at 30× magnification; CT scan images in single plane (**e**) and 3D reconstruction (**f**).

The bioinks were printed using a RFP bioprinter [38] to obtain structures with flexible and customized geometry. The design of the scaffold was carried out in SolidWorks2015 (Solidsolution, London, UK) and the slicing process was implemented with customized Slic3r version 1.3.0.64bit open-source software (https://slic3r.org/ (accessed on 18 September 2023)). An Alg/Gel scaffold digital model was created with dimensions of 16 × 16 mm (six layers, 0.2 mm layer height), featuring a specific reticular internal geometry (fill density 40%), obtained either in the presence (Alg/Gel+apoA-I) or absence (Alg/Gel) of apolipoprotein A-I. The scaffolds were printed with a 25 G nozzle (Chemil s.r.l., Padova, Italy) and 5ml syringe (Chemil s.r.l., Padova, Italy) at room temperature (RT) at 25 mm/s of printing speed, directly onto an aluminum plate displaying a temperature above −15 °C. This setup allowed the bioink to freeze instantly upon deposition, enabling immediate physical crosslinking of the gelatin component. After bioink deposition and scaffold formation (Figure 1a), a combined gelation process occurred using 50 mM Calcium Chloride (CaCl_2_) as a gelling agent.

In detail, CaCl_2_ was added slowly on the scaffold, to ensure the preservation of the structural integrity, and preserved for 1 min at the same freezing temperature of the bioink to reach the complete crosslinking of the material (Figure 1b,c), as already shown in previous studies [38,39]. High resolution X-ray tomography (Cheetah EVO system, Comet Yxlon GmbH, Wunstorf, Germany) was performed (CT settings: 60 kV, 270 uA) to study the 3D structure of the scaffold.

### 2.3. Rheological and Mechanical Tests

The rheological properties of the bioink were tested with the use of a viscosimeter (PCE-RVI 2, PCE Instruments, Capannori (LU), Italy) at three experimental temperatures, namely 25 °C, 30 °C, and 37 °C. All tests were repeated at least three times.

In parallel, rectangular specimens with a nominal thickness of 1.3 mm were excised from the fabricated films to assess the mechanical properties of the matrix. These specimens were then secured within a custom-engineered load cell [40,41] housed in a climatic chamber maintained at 25 or 37 °C and 100% RH, where they were subjected to a sinusoidal oscillatory load. To prevent grip slippage and ensure the matrix remained within its elastic limit, the applied displacement was restricted to a minimal amplitude of approximately 60 µm, corresponding to a strain of about ±1%. In parallel, a separate set of specimens underwent indentation testing using a wedge indenter, allowing for the determination of the fracture point of the matrix under compressive loading.

### 2.4. Dissolution Test

A qualitative dissolution study was performed to assess the structural stability of the scaffolds under simulated physiological conditions. Alg/Gel scaffolds, after the bioprinting process, were immersed in a 50 mM CaCl_2_ solution for varying durations (5 min, 15 min, 30 min, 1 h, and 2 h) to evaluate the structural changes resulting from different crosslinking times.

**Figure 2:**
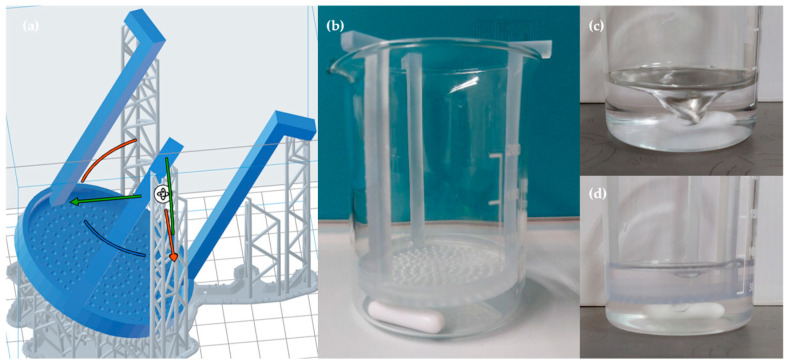
(**a**) Pre-designed perforated resin structure suitable for the beaker used in the dissolution test; (**b**) beaker with perforated resin support and magnetic stir bar before the dissolution test; (**c**) beaker before inserting the resin structure subjected to a 200-rpm stirring motion; (**d**) beaker after inserting the perforated resin structure, subjected to a 200-rpm stirring motion.

After crosslinking, each scaffold was divided into four equal parts. Three of the four parts were placed on a perforated resin grid and immersed in high-glucose DMEM (DMEM High Glucose, Dominique Dutscher, Bernolsheim, France) contained in a 250 mL glass beaker (Figure 2b). The beaker was positioned on a Heating Magnetic Stirrer (AREX Digital Pro, VELP Scientifica, Usmate Velate (MB), Italy) set at a constant temperature of 37 °C to mimic physiological conditions. The medium was kept in continuous motion at 200 rpm using a magnetic stir bar to simulate dynamic flow conditions (Figure 2c,d).

Scaffold samples were retrieved at different time points (15 min, 30 min, and 45 min) to assess structural changes, handling characteristics, and the extent of dissolution under these dynamic and thermally controlled conditions.

### 2.5. In Vitro Biological Testing

#### 2.5.1. Cell Cultures and Samples Preparation

In vitro cellular assays related to the biocompatibility of the obtained scaffolds were performed using human endothelial cells (HUVEC) obtained from the American Type Culture Collection (ATCC, distributed from LGC Standards srl, Milan, Italy). Cells were cultured in complete Endothelial Cells Basal Medium 2 (EBM-2—Lonza, Walkersville, MD, USA) supplemented with the specific endothelial stimuli (EGM-2 Single Quots, Lonza) and kept in a humidified atmosphere at 37 °C with 5%ppCO_2_.

The release of apoA-I into the culturing medium was observed with the use of murine macrophages cell line (J774) obtained from the American Type Culture Collection (ATCC, distributed from LGC Standards srl).

Macrophages were cultured in DMEM High Glucose (Dominique Dutscher, Bernolsheim, France), addictionated with 1% Penicillin/Streptomycin (Pen/Strep, Corning, Mediatech Inc., Manassas, VA, USA) and 10% Fetal Bovine Serum (FBS, Dominique Dutscher, Bernolsheim, France), and kept in a humidified atmosphere at 37 °C with 5%ppCO_2_.

Before proceeding to the in vitro testing phase, 3D-printed scaffolds were UV irradiated for 20 min. HUVEC cells were then detached from the substrate with trypsin 0.25% (25-053-CI, Corning) and seeded at a density of 5 × 10^4^ cells/mL for the metabolic/viability evaluation, and at 1 × 10^5^ cells/mL for the fluorescent staining assay. In the same way, J774 macrophages were detached from the surface and seeded at a density of 1.5 × 10^5^ cells/mL directly onto the TCPS 48-multi-well for the efflux experiments. All the experiments presented a control group of cells not treated with apoA-I and in the absence of the biomaterials.

#### 2.5.2. Cholesterol Efflux Assay

The release of active apoA-I from the 2% Alg/10%Gel scaffolds has been studied through its effectiveness in the regards of cholesterol release and through the development of a cholesterol efflux assay on J774 murine macrophages starting from different concentrations of protein, namely: 0 µg/mL, 2.5 µg/mL, 5 µg/mL, and 10 µg/mL.

The efflux assay is designed to quantitate the rate of cholesterol efflux from cultured cells as extensively reported in the literature and as follows [42]: initially, J774 cells were plated as previously described, labeled with 2μCi 3[H]-cholesterol for 24 h in medium with 1% FCS and 2 µg/mL of an ACAT inhibitor (Sandoz 58035, Sigma-Aldrich) to ensure that all labeled cholesterol remained as free cholesterol.

Cells were then allowed to equilibrate by incubation for 18 h with 0.2% BSA. After this, some wells were washed with PBS, dried, and extracted with 2-propanol to provide baseline (time 0) values for total cell [3H]-cholesterol content. Remaining cells were washed with PBS and incubated with Alg/Gel scaffolds, Alg/Gel+apoA-I (2.5 µg/mL, 5 µg/mL and 10 µg/mL) scaffolds, and pure apoA-I (2.5 µg/mL, 5 µg/mL and 10 µg/mL). All the experimental groups were treated in the presence and in the absence of 0.3mM of cAMP—to evaluate the efflux process mediated by the transporter ABCA1 and by passive diffusion, respectively. Subsequently, supernatants were filtered through a 0.45 µm filter to remove floating cells, and radioactivity was determined by liquid scintillation counting in supernatants and in time 0 cell extracts. The rate of incorporated/released cholesterol was quantified with the following formula: (cpm in medium at 4h/cpm at time 0) × 100. Finally, the evaluation of ABCA1 mediate efflux was determined as the difference of the efflux of the group stimulated with cAMP and the control group.

#### 2.5.3. Cytotoxicity Evaluation

To observe the biological response of human endothelial cells when posed in direct contact with a culturing medium containing the biomaterials, the Alg/Gel scaffold with the higher percentage of Apolipoprotein A-I (10 µg/mL) has been selected as test group, while the Alg/Gel scaffold without ApoA-I and the cells cultured onto the plastic substrate have been used as controls. Validated tests for cellular metabolic activity and viability have been performed at the experimental time point of 24, 48, 72, 168, and 240 h after cell seeding. Cell metabolic activity was initially investigated through an MTT assay (Roche Diagnostics GmbH, Mannheim, Germany) and cell counting. Briefly, during MTT assay, culture supernatant was removed from the plate and 100 µL of pristine medium diluted with 10% of MTT labeling reagent was added to the plate and incubated at 37 °C for 4 h. Subsequently, 100 µL of solubilization solution was added to each well and specimens were incubated overnight in standard culturing conditions. At the term of the incubation, the absorbance of the formazan product was quantified at 620 nm with a Multiskan^®^ FC microplate reader (Thermo Fisher Scientific).

Cellular proliferation rate was analyzed through a chemiluminescent assay (CellTiter-GLO, Promega, Madison, WI, USA) performed to assess cell viability through the quantitation of the ATP present in the samples. Briefly, a culture medium was discarded, and a 50:50 solution of CellTiter-Glo lysis buffer and complete culture medium was added. Each sample was incubated for 2 min on an orbital shaker, the solution was collected, and then luminescence stabilized for 10 min in the dark. Samples were then centrifuged for 30 s to eliminate any bubbles, and luminescence was measured with a luminometer with double injectors (GloMax 20/20, Promega). Finally, to evaluate the precise number of cells present in each experimental well, cells were detached from the surface, diluted in 1ml of pristine medium, diluted with Trypan Blue (Gibco) and counted with a Burker’s Chamber. Prior to performing all the aforementioned tests, HUVEC cells adherent to the plastic substrate were observed during the culturing time in contact with the biomaterials with an inverted microscope (Nikon Eclipse TS100, Nikon, Tokyo, Japan) and images were taken to show their morphology and disposition, besides corroborating the results of the biological tests.

Finally, to observe cell morphology and evaluate the viable/dead cell rate, a fluorescent staining was performed at 24 h with Calcein AM (Sigma-Aldrich, St. Louis, MI, USA) and Propidium Iodide (Sigma-Aldrich, St. Louis, MI, USA). Briefly, supernatant was discarded, cells were washed in Phosphate Buffer Saline (PBS, SigmaAldrich, St. Louis, MI, USA) and subsequently incubated with Calcein-AM 4 µM and Propidium Iodide 4,5 µM in PBS for 15 min at room temperature in dark conditions. After extensive washing in PBS, specimens were mounted under glass cover slips with a mounting medium (Dako Cytomation Fluorescence Medium, Agilent Technologies, Santa Clara, CA, USA) for photo bleaching reduction. Samples were observed with a fluorescence microscope Zeiss Axio Imager A.2 (Carl Zeiss, Jena, Germany) using a 10X objective. The images were analyzed with ImageJ software (version 1.54p, National Institutes of Health, Bethesda, MD, USA) to obtain the ratio of living/dead cells on all the samples.

### 2.6. Statistical Analysis

Data was analyzed using Prism 8 (GraphPad Software, San Diego, CA, USA). All the values were reported as mean ± standard deviation of at least three repeated measurements. After the initial validation of the normal distribution of the data through the Shapiro–Wilk test, the differences amongst groups were evaluated with 2-way analyses of variance. Then, the Tukey post-test was applied for multiple comparisons. Differences were considered statistically significant when *p* < 0.05.

## 3. Results

### 3.1. Rheological and Mechanical Characterization

The rheological properties of the bioink, as well as the settings of the analysis, are presented in Table 1.

The viscosity measurements demonstrate the rheological behavior of the sample at three different temperatures using appropriate rotor geometries and rotational speeds. A clear inverse correlation between temperature and viscosity was observed. At 25 °C, the sample exhibited the highest viscosity (5000 ± 100 cP), which decreased to 2800 ± 100 cP at 30 °C and further declined to 460 ± 20 cP at 37 °C, which is the printing temperature of the bioink. This trend is consistent with the behavior of polymeric or semi-solid systems, in which increasing temperature reduces intermolecular interactions, thereby lowering viscosity. Obtained data suggest that the material exhibits rheological characteristics typical of gels, emulsions, or polymer dispersions—systems known for their shear-dependent behavior and structural integrity. Such properties are critical for applications in 3D printing and biomedical engineering, where precise control of flow and stability is essential.

**Figure 3:**
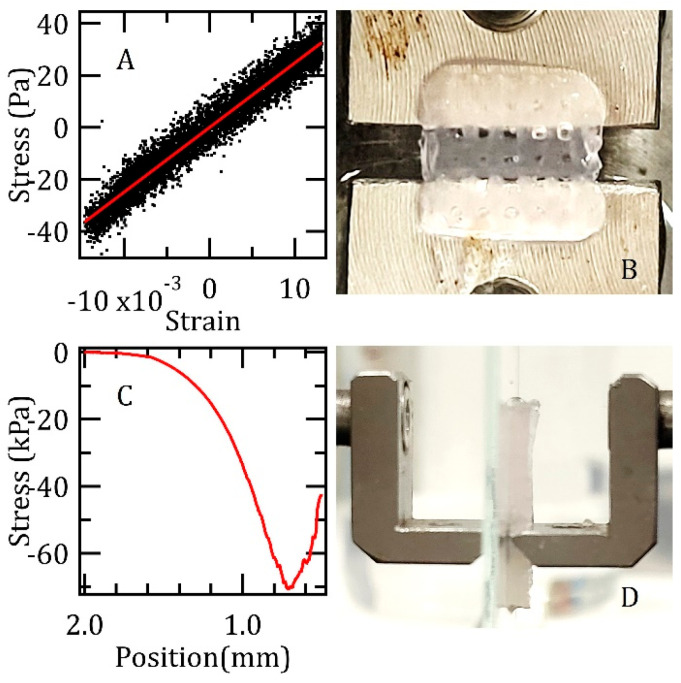
(**A**) illustrates a representative stress–strain curve obtained from a specimen subjected to a sinusoidal loading regime at room temperature; (**B**) depicts the specimen, with a measured width of approximately 5.7 mm, prior to its securement within the clamps of the testing apparatus; (**C**) presents a typical load curve resulting from an indentation test at room temperature; (**D**) displays the relevant specimen, possessing a nominal thickness of 1.3 mm, positioned between a slide and the wedge during the indentation testing procedure.

The fabricated Alg/Gel scaffolds, gelled with 50 mM CaCl_2_, were evaluated using a load cell. Preliminary results from the mechanical characterization conducted at room temperature are shown in Figure 3, which also includes photographs illustrating the experimental setup. The upper panels show an example stress–strain curve, derived from sinusoidal loading, which yielded a Young’s modulus of (2.5 ± 0.3) kPa. The lower panels detail indentation tests performed with a wedge indenter, revealing breakpoint stress values of approximately (70 ± 10) kPa.

Consistent with the values reported in Table 2, testing the specimens at the physiological temperature of 37 °C again yielded a Young’s modulus of (2.5 ± 0.3) kPa. The measurement showed excellent reproducibility, with the stated error originating from the inaccuracy in determining the specimen cross-section, not from sample-to-sample variations. Under X-ray tomography, no morphological changes were discernible, confirming that the low applied strains kept the samples within their elastic regime. However, in contrast to the room temperature results, compressive loading at 37 °C did not lead to a clear fracture point. Instead, the samples underwent continuous thinning accompanied by minor fracturing.

### 3.2. Dissolution Test

**Figure 4:**
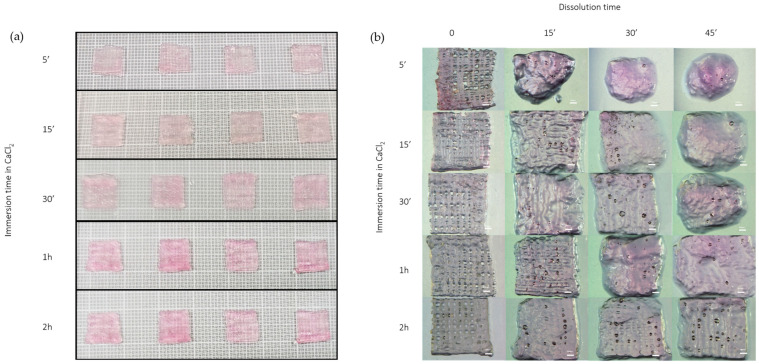
(**a**) scaffolds before dissolution tests at different immersion times in CaCl_2_ (5 min, 15 min, 30 min, 1 h, 2 h); (**b**) scaffolds at different immersion times in CaCl_2_ (5 min, 15 min, 30 min, 1 h, 2 h) and dissolution time (0 min, 15 min, 30 min, 45 min) under stereomicroscope at 10× magnification.

The dissolution test confirmed that crosslinking time plays a crucial role in determining scaffold stability. As illustrated in Figure 4, shorter immersion times in the CaCl₂ crosslinking solution resulted in a faster dissolution rate and a more evident loss of shape and structural integrity. In contrast, scaffolds crosslinked for 2 h exhibited optimal mechanical stability and maintained their architecture long enough to allow for in vitro testing. These results highlight that a 2-h crosslinking duration offers an effective balance between structural integrity and controlled degradability, making it suitable for subsequent biological applications.

### 3.3. Cholesterol Efflux Assay

**Figure 5:**
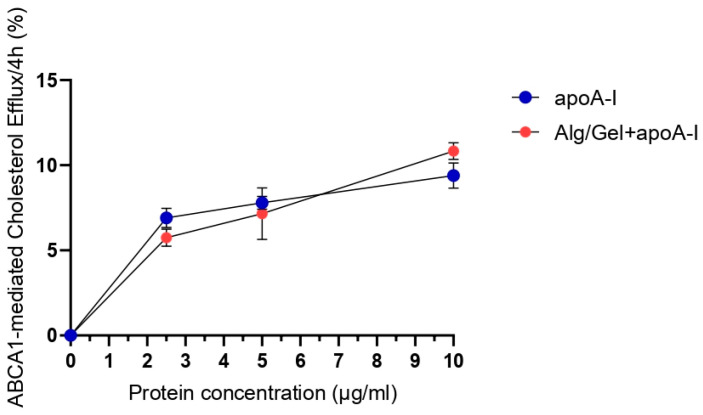
Results of ABCA1-mediated cholesterol efflux after 4h of analysis. J774 cells were stimulated with cAMP and Alg/Gel+apoA-I scaffolds with 0 µg/mL, 2.5 µg/mL, 5 µg/mL, and 10 µg/mL compared to the same concentration of free apoA-I not complexed with the biomaterial. Basal condition effect (not stimulated with cAMP) has already been subtracted to the shown data.

The possibility of the 3D-printed scaffold to release different amounts of apolipoprotein A-I in the culturing medium, and in an active and stable form, has been studied through a cholesterol efflux assay. Scaffolds comprising 2% Alg and 10% Gel, with increasing concentrations of apoA-I (0 µg/mL, 2.5 µg/mL, 5 µg/mL, and 10 µg/mL), were analyzed in direct contact with J774 murine macrophages. Figure 5 shows the release rate of cholesterol in the presence of cAMP stimuli.

The data obtained underline the lack of statistically significant differences between the efflux mediated by the free protein apoA-I and the protein released by the 3D-printed structure. The experimental groups underwent to basal conditions (not stimulated with cAMP) at all the tested concentrations of free or complexed apoA-I, showing the same pattern of efflux and a medium value of about zero, as corroborated from the literature [11].

### 3.4. Cytotoxicity Evaluation

**Figure 6:**
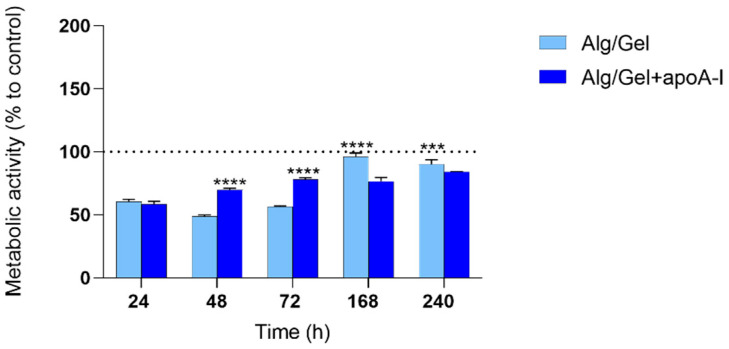
MTT metabolic activity evaluation on Alg/Gel and Alg/Gel+apoA-I scaffolds in contact with HUVEC cells. The normalization value of the control group is settled at 100% (dotted line). **** *p* < 0.0001 Alg/Gel vs. Alg/Gel+apoA-I and *** *p* = 0.0001 Alg/Gel vs. Alg/Gel+apoA-I.

The preliminary in vitro analysis of human endothelial cells cellular behavior, in regards to Alg/Gel and Alg/Gel+apoA-I (10 µg/mL) scaffolds, underlined their non-cytotoxicity. In detail, Figure 6 shows the metabolic activity of HUVEC cells grown in contact with the biomaterials at different time points; the metabolic activity of each experimental group has been normalized to the control group (100%). As shown in the graph, up to 72 h, the metabolic activity of Alg/Gel and Alg/Gel+apoA-I groups were slightly lower when compared to the control group, but tended to increase during the time, reaching the same level 7 days after seeding in the Alg/Gel group. Noteworthy, there is quite a linear pattern in the values related to Alg/Gel+apoA-I scaffolds during the time, shifting from values higher than those of Alg/Gel scaffolds (at 48 and 72 h *p* < 0.0001 Alg/Gel+apoA-I vs. Alg/Gel) to lower values at 7 and 10 days (*p* < 0.0001 at 7 days and *p* = 0.0001 at 10 days Alg/Gel vs. Alg/Gel+apoA-I). Nevertheless, in the intra-group analysis of the single groups Alg/Gel and Alg/Gel+apoA-I, a constant statistically significant increase of metabolic activity over the time is present, except for Alg/Gel+apoA-I at 72 h vs. 168 h.

**Figure 7:**
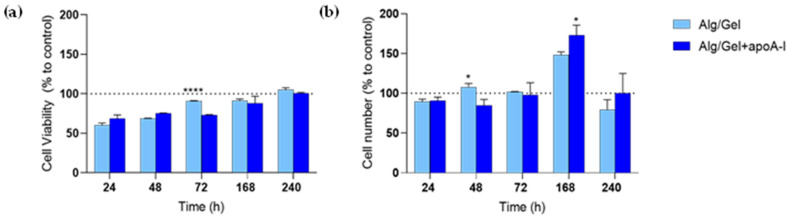
(**a**) CellTiter-GLO chemiluminescent assay on Alg/Gel and Alg/Gel+apoA-I scaffolds in contact with HUVEC cells. **** *p* < 0.0001 Alg/Gel vs. Alg/Gel+apoA-I. (**b**) Quantification of HUVEC cells cultured with Alg/Gel and Alg/Gel+apoA-I scaffolds. * *p* = 0.0391 Alg/Gel vs. Alg/Gel+apoA-I at 48 h and * *p* = 0.0215 Alg/Gel+apoA-I vs. Alg/Gel at 168 h. All the values have been normalized to the value of control group which is settled at 100% (dotted line).

In parallel, the cells’ proliferation rate, evaluated as the variation of ATP production in the samples has been quantified (Figure 7a), as well as HUVEC cells number into the experimental wells (Figure 7b).

CellTiter-GLO showed the same pattern seen in the metabolic activity assay; at 24 and 48 h it seems to be lower than the control group, but it sharply increases reaching the 72 h of culture. The viability rate of HUVEC cells seems to be higher in Alg/Gel group when compared to control and Alg/Gel+apoA-I, but no statistically significant differences were found comparing the two groups. On the contrary, the intra-group analysis of Alg/Gel+apoA-I samples underlined statistically significant differences at 72 h vs. 168 h (*p* < 0.0001) and at 168 h vs. 240 h (*p* = 0.0012), confirming the increase of ATP production during the time.

The increase observed in cell viability is corroborated by the count of the adhered cells present in the single wells (Figure 7b) where the constant increase of vital cells in both the experimental groups is confirmed (*p* < 0.0001 Alg/Gel 72 h vs. 168 h and 168 h vs. 240 h and Alg/Gel+apoA-I 72 h vs. 168 h and 168 h vs. 240 h). A slight decrease of vital cells seen at 10 days after seeding should be highlighted, which could be most likely related to the confluence reached by the cells and to the long culturing time.

**Figure 8:**
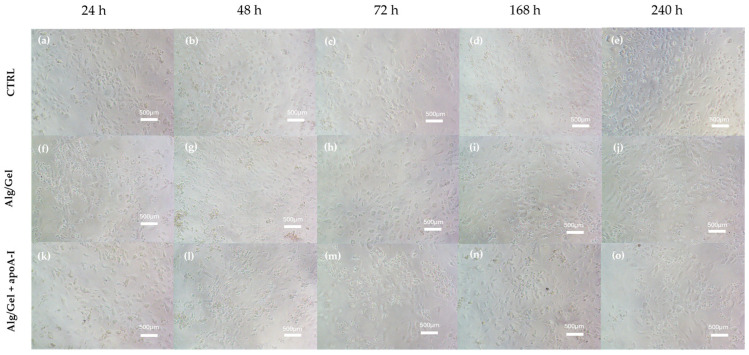
Cellular morphology observed through optical microscopy of HUVEC cells cultured for the experimental time points of 24–48–72–168–240 h; (**a**–**e**) control group; (**f**–**j**) Alg/Gel group; and (**k**–**o**) Alg/Gel+apoA-I group.

HUVEC morphology when cultured with a medium containing the biomaterials has been evaluated by light microscopy and live/dead assay. Figure 8 shows the morphology of adhered cells in all the experimental conditions at all the selected time points.

**Figure 9:**
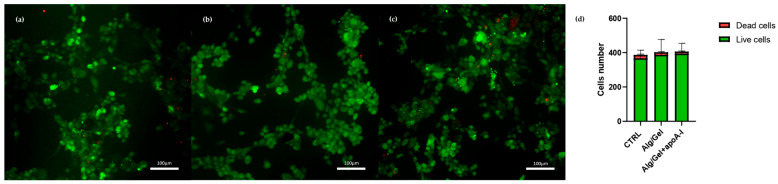
Calcein-AM and Propidium Iodide straining on HUVEC cells at 24 h. (**a**) Control group; (**b**) HUVEC cells in the presence of medium conditioned with Alg/Gel scaffold; (**c**) HUVEC cells in the presence of medium conditioned with Alg/Gel+apo A-I scaffold; and (**d**) quantitative evaluation of living and dead cells ratio.

HUVEC presenting the typical polygonal shape indicates a healthy state. The images underline the lack of cytotoxic effects due to the presence of the biomaterials into the culturing medium, allowing to hypostatize a correct interaction of the biological part with alginate, gelatin, and apolipoprotein A-I. This aspect further corroborates the images obtained through viability evaluation with Calcein-AM and Propidium Iodide (Figure 9). Calcein-AM and Propidium Iodide staining revealed that cell viability after 24 h was not affected by the presence of the biomaterial either in the presence or absence of ApoA-I. As shown in Figure 9, HUVEC cells cultured in the presence of Alg/Gel and Alg/Gel+apoA-I samples show good cell viability, confirming an active metabolic state on all the tested groups. Moreover, the quantitative analysis of live and dead cells (Figure 9d) on all the samples showed no statistically significant differences among the studied groups, confirming that the percentage of dead cells was a minority of the total.

## 4. Discussion

Alginate-based bioinks, widely studied in the literature, have demonstrated various potential advantages in relation to the printing process. Specifically, the RFP-based fabrication method [26] has shown that the selected formulation can achieve gelation in just 5 min with CaCl_2_ concentrations below 1% (50 mM), a lower value than typically reported in the literature [27]. Indeed, a low concentration of CaCl_2_ allows the structure to create less stable crosslinking thus obtaining a biomaterial able to rapidly dissolve into the microenvironment, acting as a suitable construct for fast drug/protein release [43]. This is made possible by the porosity generated during the freezing process [38], which significantly increases the surface area available for interaction with the gelling agent. Our results may provide the foundation for developing a highly customizable coating for DCB, utilizing innovative materials tailored to meet specific therapeutic needs.

Concerning the mechanical properties of the system, the literature values for the Young’s moduli of alginate-based bioprinted scaffolds vary significantly, spanning several orders of magnitude from a few kPa or less [44] to approximately 10 kPa [45,46], reaching 100 kPa [47], and even up to 1 MPa [48]. Compared to previous reports, our relatively low value of (2.5 ± 0.3) kPa is likely a result of the combined effects of the unique mesh structure of the matrix, the low concentration of bivalent cation crosslinkers, and the high gelatin content [47,49]. Hysteresis, clearly observed during the measurements, is attributed to potential sample slippage within the grips, which were carefully engaged to minimize matrix degradation, and this may have introduced a small systematic error. The breakpoint stress behavior under wedge indentation, which exhibited a spectrum of responses from rupture at approximately (70 ± 10) kPa at room temperature to continuous thinning under physiological conditions, is consistent with the mechanical demands for integration within a stent balloon inflated at pressures below 1 Atm. While our films are robust enough for manipulation, their mechanical properties categorize them as highly fragile. This fragility is acceptable, and even advantageous, for their intended use in carrying and delivering a drug payload to the vessel walls during surgical procedures.

These results, when compared with data available in the literature, help to contextualize the mechanical behavior of the developed scaffold. Alginate-based coatings are widely studied for biomedical uses, especially in drug-coated balloon (DCB) technologies, due to their adaptable mechanical characteristics and gelation properties/ionic crosslinking [50]. Moreover, the obtained differences, likely due to the reduced CaCl_2_ concentration and the relatively high gelatin content in our system have to be used in order to fit the described literature range of 60–80 kPa [51]. In fact, the selection of coating materials in DCB design plays a crucial role in balancing mechanical compliance, drug delivery efficiency, and biological performance. Natural polymer coatings such as hyaluronic acid, frequently employed in vascular and soft tissue applications, offer excellent biocompatibility and resorption profiles but generally exhibit lower mechanical stiffness, with Young’s modulus values typically ranging from 1 to 3 kPa [52]. In contrast, chitosan-based systems provide increased mechanical robustness (up to 15–20 kPa) [53], though they may lack the elasticity necessary for compliance with dynamic vascular environments. On the other hand, synthetic polymer coatings such as PLGA and PCL are widely adopted in commercial DCBs due to their high mechanical stability and programmable degradation rates. These materials often demonstrate Young’s modulus values in the MPa range [54], which, while beneficial for drug retention and long-term structural integrity, may result in stiffness mismatches with native tissue and reduced vascular compatibility. Moreover, synthetic coatings often require surfactants or hydrophilic additives (e.g., PEG, iopromide) to modulate drug release and biointeraction. Compared to these systems, our alginate/gelatine scaffold offers a mechanically compliant interface that is well-aligned with the properties of coating used for soft vascular tissues. Its relatively low modulus and rupture strength remain within the range reported for bioinspired DCB coatings, while providing additional advantages such as enhanced porosity, tunable degradation, and rapid-release capability. These features are especially desirable for temporary vascular applications where high conformability and efficient drug delivery are required. Thus, the proposed formulation represents a promising alternative to both natural and synthetic systems currently in use or under investigation in DCB technologies.

Nonetheless, the recent literature highlights the necessity of moving beyond traditional drug/excipient formulations toward a more integrated understanding of the biophysical mechanisms underlying DCB performance [51]. In alignment with this view, our study included both mechanical testing of the scaffold at physiological temperature (37 °C) and rheological analysis of the bioink at various temperatures (25 °C, 30 °C, and 37 °C). This strategy enabled us to simulate both pre-deployment and in situ conditions, providing valuable insights into how temperature-sensitive properties may influence coating adhesion, mechanical compliance, and functional behavior during balloon expansion. However, given the complex and dynamic nature of the vascular environment, we recognize that in vitro models alone are insufficient to fully reproduce these interactions. For this reason, we are currently designing in vivo experiments to further evaluate the coating’s behavior under realistic physiological conditions. This comprehensive approach reinforces the translational relevance of our work and directly responds to key challenges identified in the evolving field of DCB technologies.

Considering that the alginate enable the management and fast release of filling agents (i.e., apolipoprotein A-I) [55], to evaluate the ability of the structure to release a biological filler, the efflux assay was performed on three different concentrations of apoA-I (2.5, 5, 10 µg/mL) incorporated in the scaffolds. As shown in Figure 6, the ability to perform even in vivo conditions for cholesterol efflux was confirmed by the effectiveness of active protein release from the 3D structure. Indeed, cholesterol efflux after 4 h of testing was comparable from Alg/Gel scaffolds implemented with the protein and the free protein (control group) at all the tested concentrations. This specific result has been obtained in the cAMP stimulated group, expressing ABCA1 protein, with the same pattern and with a ratio of about ten times more when compared to the basal non-stimulated one, which can be expected, since apoA-I is a specific cholesterol acceptor for ABCA1 membrane protein [13]. Assuming that there is no difference in the relative release of apoA-I from scaffolds doped with the three concentrations named above, the higher (10 µg/mL) concentration of apoA-I has been selected for the biological testing of the scaffold to assess the material as suitable for clinical applications.

On the biological counterpart, the cytotoxicity analysis showed a not cytotoxic behavior for all the tested structures (Alg/Gel and Alg/Gel+apoA-I) and at all the experimental time points (24, 48, 72, 168, and 240 h). Cellular morphology of HUVEC cells was predominantly polygonal and adhered to the surface, index of healthy cells, even after 7–10 days of culture. Noteworthy, on the Alg/Gel and Alg/Gel+apoA-I scaffolds, the cellular shape was slightly more rounded, probably due to their effective adhesion to the hydrogel structure that induces a more circular morphology [14]. This is also confirmed by Calcein-AM staining that revealed great cell viability after 24 h, which was not affected by the presence of the biomaterial and was comparable among the treated scaffolds and the control group. Turning on the metabolic activity and viability analysis (Figure 6 and Figure 7), the complementarity of the data obtained is noteworthy. Indeed, in the MTT assay, even if the data are always lower when compared to the control (100%—not treated cells), the value obtained from cells treated with Alg/Gel+apoA-I scaffolds, at 48 h, reached the level of 70%, considered not cytotoxic as per the ISO 10993-5 guidelines on the cytotoxicity of porous materials [56]; additionally, the index of adaptation of the cells to the hydrogel probably remains stable on the long-term time points (*p* < 0.0001 Alg/Gel+apoA-I vs. Alg/Gel at 48 h and 72 h). It is interesting to note that the same value on the Alg/Gel has been reached only after 7 days of culture, probably due to the longer time needed for the adaptation of HUVEC in the absence of apoA-I, but after the adaptation period, it tends to increase at 7 and 10 days (*p* < 0.0001 Alg/Gel vs. Alg/Gel+apoA-I at 168 h and 240 h). In this regard, different research groups underlined that when apoA-I is bonded by endothelial cells, it induces the overexpression of Ciclooxygenase-2 and the release of Prostaglandin E2 through ABCA1, an effect that could partially mediate the antiatherogenic effect of HDL in vivo, but at the same time could enhance the metabolic activity of HUVEC at short time points [57,58].

This metabolic result can be interestingly correlated to the viability test and to the cell counting evaluation; indeed, considering ATP production and the number of cells in the samples, there is a constant intra-group increase of ATP synthesis, as well as cell number, that reaches the control value after 10 and 7 days of culture, respectively. The result of ATP production seems to support the idea that there might be an initial adaptation time ranging from the seeding day to day 3 that allows the cells to adapt to the hydrogel presence and then allows them to actively proliferate. All the data of cell counting (Figure 7b) correlate in a linear way with the ATP production, corroborating the idea that the biomaterial is not cytotoxic for up to ten days of culture. Interestingly, cell number also seems to have a latency time at 48 h, where the Alg/Gel+apoA-I group shows less cells when compared to Alg/Gel (*p* = 0.0391); however, it sharply increases again after 7 days (*p* = 0.0215). Additionally, the hydrogel presence might provide a suitable environment that does not impair cellular proliferation and biological activity, and at the same time, the presence of apolipoprotein A-I may enhance the biological activity of HUVEC cells [56,57,58,59].

## 5. Conclusions

The preliminary data obtained in this study confirm that RFP bioprinting technology can assure high resolution and the ability to manage with accuracy Alg/Gel scaffolds enriched with high-density lipoproteins, that preserve their activity and are not damaged by the printing process. Our findings can be applied to create customizable coatings for DCB in the foreseeable future. Indeed, the cholesterol efflux analysis underlined that all the selected concentrations of apoA-I have been efficiently released by the 3D structure, showing comparable activity between the 3D-printed scaffolds and the free protein. Moreover, Alg/Gel scaffolds both in the presence and in the absence of 10 µg/mL of apolipoprotein A-I are biocompatible in the regards of human endothelial cells (HUVEC). This makes our Alg/Gel+apoA-I scaffold a suitable candidate for future bioactive agents’ delivery applications in vascular surgery, that can be personalized when encountering specific patients’ needs.

## Figures and Tables

**Table 1:** Rheological properties of the bioink at different temperatures (25 °C, 30 °C, and 37 °C).

	25 °C	30 °C	37 °C
Rotor	R6	R6	R3
RPM	100	200	100
Tolerance interval (%)	51.5–52.5	56–58	47
cP (mPa)	5000 °C ± 100	2800 °C ± 100	460 °C ± 20

**Table 2:** Stress–strain curve analysis performed at 37 °C and 100% RH in a climatic chamber.

Specimen Nr	1	2	3
Young’s Modulus (KPa)	2.51 ± 0.03	2.50 ± 0.05	2.57 ± 0.04

## Data Availability

The data that support the findings of this study are available from the corresponding author upon reasonable request.
